# Structure of fructose bisphosphate aldolase from *Bartonella henselae* bound to fructose 1,6-bisphosphate

**DOI:** 10.1107/S174430911101894X

**Published:** 2011-08-13

**Authors:** Anna Gardberg, Jan Abendroth, Janhavi Bhandari, Banumathi Sankaran, Bart Staker

**Affiliations:** a Emerald BioStructures, 7869 NE Day Road West, Bainbridge Island, WA 98110, USA; b Department of Allergy and Infectious Diseases, School of Medicine, University of Washington, Seattle, Washington USA; c Advanced Light Source, USA

**Keywords:** SSGCID, fructose bisphosphate aldoloses, *Bartonella henselae*

## Abstract

Fructose bisphosphate aldolase (FBPA) enzymes have been found in a broad range of eukaryotic and prokaryotic organisms. FBPA catalyses the cleavage of fructose 1,6-bisphosphate into glyceraldehyde 3-phosphate and dihydroxy­acetone phosphate. The SSGCID has reported several FBPA structures from pathogenic sources, including the bacterium *Brucella melitensis* and the protozoan *Babesia bovis*. Bioinformatic analysis of the *Bartonella henselae* genome revealed an FBPA homolog. The *B. henselae* FBPA enzyme was recombinantly expressed and purified for X-ray crystallographic studies. The purified enzyme crystallized in the apo form but failed to diffract; however, well diffracting crystals could be obtained by cocrystallization in the presence of the native substrate fructose 1,6-bisphosphate. A data set to 2.35 Å resolution was collected from a single crystal at 100 K. The crystal belonged to the orthorhombic space group *P*2_1_2_1_2_1_, with unit-cell parameters *a* = 72.39, *b* = 127.71, *c* = 157.63 Å. The structure was refined to a final free *R* factor of 22.2%. The structure shares the typical barrel tertiary structure and tetrameric quaternary structure reported for previous FBPA structures and exhibits the same Schiff base in the active site.

## Abstract 2

While other aldolases crystallize readily in the apo form, diffraction-quality crystals of *B. henselae* aldolase could only be obtained in the presence of the native substrate. The quaternary structure is tetrameric, as is typical of aldolases.

## 1. Introduction

### 1.1. SSGCID

The Seattle Structural Genomics Center for Infectious Disease (SSGCID) is one of two consortia funded by NIAID to apply genome-scale approaches to the solution of protein structures from bio­defense organisms, as well as those causing emerging and re-emerging diseases. In its first three and a half years, the SSGCID has submitted ∼350 protein structures to the Protein Data Bank (PDB) and is on track to solve a further 100 per year going forward. For several organisms, this represents the majority of PDB submissions during this time, including 100% of the structures for *Ehrlichia*, *Anaplasma* and *Burkholderia*. SSGCID’s target-selection strategy has focused on drug targets, essential enzymes, virulence factors and vaccine candidates from a number of bacterial (*Bartonella*, *Brucella*, *Ehrlichia*, *Anaplasma*, *Rickettsia*, *Burkholderia*, *Borrelia* and *Mycobacterium*) and eukaryotic (*Babesia*, *Cryptosporidium*, *Toxoplasma*, *Giardia*, *Entamoeba*, *Coccidioides* and *Encephalitozoon*) pathogens, as well as ssDNA and negative-strand ssRNA viruses. More than 3000 targets have been selected to date, with >700 proteins being purified for crystallization trials. Crystallization screening and analysis of X-ray diffraction data sets for structure solution are performed at Emerald BioStructures.

### 1.2. Aldolase

Aldolases (EC 4.1.2) are enzymes that cleave aldols and have been the subject of crystallographic study since 1971 (Heidner *et al.*, 1971 ▶). To date, more than 70 unique aldolase structures have been deposited in the PDB. The glycolytic enzyme fructose bisphosphate aldolase (EC 4.1.2.13) catalyzes the aldol cleavage of fructose 1,6-bisphos­phate into dihydroxyacetone phosphate and glyceraldehyde 3-phosphate. It also catalyzes the reverse reaction. The hallmark of a class I aldolase is the presence of an active-site lysine residue which forms a Schiff base with the substrate.

*Bartonella henselae* is best known for causing cat scratch disease (CSD). It is a Gram-negative aerobic rod-shaped proteobacterium. Immunocompromised patients can develop severe complications from *B. henselae*. Here, we present the crystal structure of the class I fructose bisphosphate aldolase from *B. henselae* with the reactant bound at the active site.

## 2. Materials and methods

All expression clones, purified proteins and protein structures produced by SSGCID are available to the scientific community.

### 2.1. Protein purification and crystallization

#### 2.1.1. Purification of *B. henselae* aldolase

The protein was expressed in *Escherichia coli* using BL21 (DE3) R3 Rosetta cells and autoinduction medium in a LEX Bioreactor. Starter cultures of LB broth with appropriate antibiotics were grown for ∼18 h at 310 K as described by Choi *et al.* (2011 ▶). ZYP-5052 auto-induction medium with tryptone, yeast extract, glucose and α-lactose supplemented with MgSO_4_ and trace metals was freshly prepared as per Studier’s published protocol (Studier, 2005 ▶). Antibiotics (carbenicillin/ampicillin) were added to 2 l bottles of sterile auto-induction medium. The medium was inoculated with all of the overnight culture. Inoculated bottles were then placed into a LEX bioreactor. Cultures were grown for ∼24 h at 298 K; the temperature was then reduced to 288 K and growth continued for a further ∼72 h. To harvest, the medium was centrifuged at 4000*g* for 20 min at 277 K. The cell paste was flash-frozen in liquid nitrogen and stored at 193 K. The frozen cells were resuspended in lysis buffer [25 m*M* HEPES pH 7.0, 500 m*M* NaCl, 5% glycerol, 30 m*M* imidazole, 0.025% sodium azide, 0.5% CHAPS, 10 m*M* MgCl_2_, 1 m*M* tris(2-carboxyethyl)phosphine (TCEP), 250 ng ml^−1^ 4-(2-aminoethyl)benzenesulfonyl fluoride hydrochloride (AEBSF) and 0.05 µg ml^−1^ lysozyme]. The resuspended cell pellet was disrupted on ice for 30 min with a Virtis sonicator (408912; 100 W power, with alternating cycles of 15 s pulse-on and 15 s pulse-off). The cell debris was incubated with 20 µl Benzonase nuclease (25 units ml^−1^) at room temperature for 45 min and clarified by centrifugation on a Sorvall SLA-1500 at 14 000 rev min^−1^ for 75 min at 277 K. The protein was purified from the clarified cell lysate by immobilized metal-affinity chromatography on a HisTrap FF 5 ml column (GE Healthcare) equilibrated with binding buffer (25 m*M* HEPES pH 7.0, 500 m*M* NaCl, 5% glycerol, 30 m*M* imidazole, 0.025% sodium azide, 1 m*M* TCEP). The recombinant protein was eluted with 250 m*M* imidazole. The purification tag (MAHHHHH­HMGTLEAQTQGPGS) was cleaved with 3C protease, leaving a GPGS remnant. The sample was further polished using size-exclusion chromatography (SEC) on a HiLoad 26/60 Superdex 75 column (GE Healthcare) in SEC buffer (25 m*M* HEPES pH 7.0, 500 m*M* NaCl, 2 m*M* DTT, 0.025% sodium azide, 5% glycerol). Fractions from a single peak were pooled. The protein was concentrated to 21 mg ml^−1^ using 10K Amicon Ultra centrifugal filters (Millipore). The concentration was determined by measuring the OD_280_ of the protein (extinction coefficient 29 450 *M* ^−1^ cm^−1^; molecular weight 39 495 Da) with a Nanodrop 1000 instrument. The concentrated sample was flash-frozen in liquid nitrogen and stored at 193 K. This purification yielded 17 mg protein per litre of cell culture with >95% purity.

#### 2.1.2. Crystallization of *B. henselae* aldolase with the native substrate fructose 1,6-bisphosphate

*B. henselae* fructose bisphos­phate aldolase (BhFBPA) was initially crystallized in the apo form, but these crystals did not diffract. Crystallization screening was repeated in the presence of the natural substrate fructose 1,6-­bis­phosphate (FBP), which yielded crystals that diffracted to 2.35 Å resolution at a synchrotron-radiation light source. BhFBPA was cocrystallized *via* vapor diffusion at 290 K in drops consisting of 0.4 µl protein solution and 0.4 µl precipitant solution, with 80 µl precipitant solution in the reservoir. Protein at 21 mg ml^−1^ in SEC buffer was supplemented with fructose 1,6-bisphosphate (FBP) from a 100 m*M* aqueous stock to give a final FBP concentration of 10 m*M* and a final protein concentration of ∼19 mg ml^−1^. A number of crystals grew, ranging in size from approximately 50 to 200 µm. The precipitant consisted of 0.2 *M* sodium acetate, 0.1 *M* Tris pH 8.5, 30% PEG 4000 (condition B10 of Crystal Screen HT from Hampton Research). The cryoprotection solution consisted of 80% precipitant solution and 20% ethylene glycol. The crystal used for data collection was soaked in this cryoprotection solution for ∼15 s before being flash-cooled in liquid nitrogen.

#### 2.1.3. Data collection, processing, structure solution and refinement

Data were collected on ALS beamline 5.0.1 as part of the Collaborative Crystallography Program over a 35 min period. The wavelength was 0.97740 Å and the detector was an ADSC Q315 CCD. The crystal-to-detector distance was 350 mm. 220 frames were collected with a width of 1° in ϕ at 100 K.

All data were indexed, integrated and scaled with the *XDS* suite (Kabsch, 2010 ▶). Data-collection and processing statistics are presented in Table 1 ▶. Refinement and validation parameters are presented in Table 2 ▶.

The structure was solved by molecular replacement with *Phaser* (McCoy *et al.*, 2007 ▶) using a search model prepared from residues 11–­343 of *Babesia bovis* aldolase (PDB entry 3kx6; SSGCID, unpublished work) with the *CCP*4*i* (Winn *et al.*, 2011 ▶) interface to *CHAINSAW* (Stein, 2008 ▶), which uses an alignment between the target sequence and that of a homologous protein of known crystal structure to create an improved molecular-replacement model by truncation of non-identical side chains. The structure was rebuilt in *Coot* (Emsley & Cowtan, 2004 ▶) and refined with *REFMAC*5 (Murshudov *et al.*, 2011 ▶) using two TLS groups per monomer. Figures were prepared with *PyMOL* (http://www.pymol.org).

## 3. Results and discussion

### 3.1. Overall structure

BhFBPA crystallized with four monomers in the asymmetric unit (50% solvent content; Matthews coefficient *V*_M_ = 2.45 Å^3^ Da^−1^; Matthews, 1968 ▶). BhFBPA adopts the typical TIM-barrel fold tertiary structure of aldolases as well as the typical homotetrameric assembly quaternary structure, with an r.m.s. difference for monomer *A* of 0.94 Å from the 3kx6 starting model. It has ∼50% sequence identity to many aldolases with unsolved structures, such as those from *Toxoplasma gondii*, *Arabidopsis thaliana* and *Crypto­sporidium parvum*. The final FBP-bound model contains four copies of FBPA including three residues of the four-residue N-terminal purification tag and residues 1–340 (of 343) of the protein; the FBP ligand was modelled in each monomer. 498 water molecules were modelled.

The final model showed good geometry (Table 2 ▶) as determined using the program *MolProbity* (Chen *et al.*, 2010 ▶).

### 3.2. Reactant state

**Figure 1:**
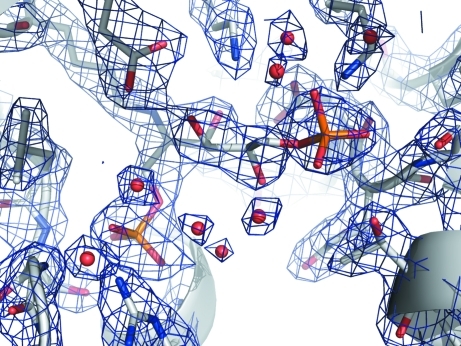
Weighted 2*F*_o_ − *F*_c_ electron-density map at 1.5σ for FBP and nearby residues at the active site of fructose bisphosphate aldolase from *B. henselae*. There is clear electron density for a Schiff base formed between Lys223 and the FBP molecule.

**Figure 2:**
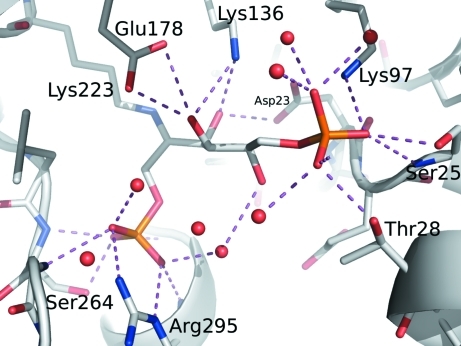
Bonding environment for FBP bound at the active site of fructose bisphosphate aldolase from *B. henselae*.

As part of glycolysis, FBPA catalyzes the cleavage of fructose 1,6-­bisphosphate (FBP) into glyceraldehyde 3-phosphate and dihydroxyacetone phosphate (DHAP). Here, we determined the 2.35 Å resolution crystal structure of BhFBPA (Tables 1 ▶ and 2 ▶). This structure has clear electron density for FBP bound at the active site (Fig. 1 ▶). There is a covalent bond between C atom C2 of the linear FBP molecule and the N^ζ^ atom of Lys223 of the protein. Furthermore, phosphate group 1 of FBP makes hydrogen bonds to three amide N atoms as well as the side chains of Arg295 and Ser264 (Fig. 2 ▶). The alcohol groups along the linear carbon backbone of FBP also make hydrogen bonds to the side chains of the protein, as well as to nearby water molecules. Finally, the O atoms of phosphate group 2 are hydrogen bonded to side chains and to nearby water molecules.

SSGCID’s interest in BhFBPA is as a potential drug target. We compared the active site of BhFBPA with those of homologous mammalian enzymes. The orientation of the FBP molecule in the active site differs from that observed in the structure of human muscle aldolase (PDB entry 4ald; Dalby *et al.*, 1999 ▶; 54% sequence identity; r.m.s.d. of non-H atoms in the FBP ligand of 2.2 Å; r.m.s.d. for C^α^ atoms over the whole protein of 0.663 Å). However, the complex observed in 4ald does not show full formation of the Schiff base, suggesting that the state in 4ald precedes the formation of the covalent complex (Dalby *et al.*, 1999 ▶). The FBP conformation in BhFBPA is essentially identical to that of rabbit muscle aldolase A (PDB entry 1zai; St-Jean *et al.*, 2005; r.m.s.d. of non-H atoms in the FBP ligand of 0.29 Å; r.m.s.d. for C^α^ atoms over a full monomer of 0.489).

## 4. Conclusion

The crystal structure of fructose bisphosphate aldolase from *B. henselae* was determined in complex with its reactant at 2.35 Å resolution. The structure obtained of the reactant Schiff-base state is similar to those of other aldolases in the reactant state [PDB entries 1zai (St-Jean *et al.*, 2005 ▶), 3mbf (Gardberg *et al.*, 2011 ▶) and 2qdg (Lafrance-Vanasse & Sygusch, 2007 ▶)], especially rabbit muscle FBPA A (PDB entry 1zai). The similarity to the active site of mammalian FBPA A suggests that the design of a specific inhibitor for BhFBPA (that does not inhibit the human enzyme) would be exceptionally challenging and would have to rely on exploiting very minor differences in the chemical environment in and near the active site.

## Supplementary Material

PDB reference: fructose bisphosphate aldolase, 3mmt

## Figures and Tables

**Table 1:** Data-collection statistics Values in parentheses are for the highest of 20 resolution shells.

Space group	*P*2_1_2_1_2_1_
Unit-cell parameters (Å)	*a* = 72.39, *b* = 127.71, *c* = 157.63
Resolution range	50–2.35 (2.41–2.35)
Unique reflections	61670 (4530)
Multiplicity	8.9 (8.4)
Completeness (%)	100 (99.0)
*R*_merge_† (%)	9.9 (55.3)
Mean *I*/σ(*I*)	19.5 (4.2)

† *R*_merge_ = 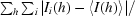 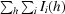.

**Table 2:** Refinement and model statistics Values in parentheses are for the highest of 20 resolution shells.

Resolution range (Å)	50–2.35 (2.41–2.35)
*R*_cryst_†	0.177 (0.233)
*R*_free_†	0.222 (0.272)
R.m.s.d. bonds (Å)	0.010
R.m.s.d. angles (°)	1.180
Protein atoms	10241
Nonprotein atoms	574
Mean *B* factor (Å^2^)	26.95
Ligand *B* factor (Å^2^)	33.2
Residues in favored region (%)	97.56
Residues in allowed region (%)	99.7
*MolProbity *score [percentile]	1.50 [99th]
PDB code	3mmt

† *R*_cryst_ = 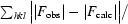 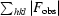. The free *R* factor was calculated using the 5% of the reflections that were omitted from the refinement.

## References

[bb4] Chen, V. B., Arendall, W. B., Headd, J. J., Keedy, D. A., Immormino, R. M., Kapral, G. J., Murray, L. W., Richardson, J. S. & Richardson, D. C. (2010). *Acta Cryst.* D**66**, 12–21. 10.1107/S0907444909042073 PMC2803126 20057044

[bb1] Choi, R., Kelley, A., Leibly, D., Nakazawa Hewitt, S., Napuli, A. & Van Voorhis, W. (2011). *Acta Cryst.* F**67**, 998–1005. 10.1107/S1744309111017374 PMC3169392 21904040

[bb3] Dalby, A., Dauter, Z. & Littlechild, J. A. (1999). *Protein Sci.* **8**, 291–297. 10.1110/ps.8.2.291 PMC2144250 10048322

[bb5] Emsley, P. & Cowtan, K. (2004). *Acta Cryst.* D**60**, 2126–2132. 10.1107/S0907444904019158 15572765

[bb15] Gardberg, A., Sankaran, B., Davies, D., Bhandari, J., Staker, B. & Stewart, L. (2011). *Acta Cryst.* F**67**, 1055–1059. 10.1107/S1744309111021841 PMC3169402 21904050

[bb6] Heidner, E. G., Weber, B. H. & Eisenberg, D. (1971). *Science*, **171**, 677–679. 10.1126/science.171.3972.677 5099718

[bb7] Kabsch, W. (2010). *Acta Cryst.* D**66**, 125–132. 10.1107/S0907444909047337 PMC2815665 20124692

[bb8] Lafrance-Vanasse, J. & Sygusch, J. (2007). *Biochemistry*, **46**, 9533–9540. 10.1021/bi700615r 17661446

[bb9] Matthews, B. W. (1968). *J. Mol. Biol.* **33**, 491–497. 10.1016/0022-2836(68)90205-2 5700707

[bb10] McCoy, A. J., Grosse-Kunstleve, R. W., Adams, P. D., Winn, M. D., Storoni, L. C. & Read, R. J. (2007). *J. Appl. Cryst.* **40**, 658–674. 10.1107/S0021889807021206 PMC2483472 19461840

[bb11] Murshudov, G. N., Skubák, P., Lebedev, A. A., Pannu, N. S., Steiner, R. A., Nicholls, R. A., Winn, M. D., Long, F. & Vagin, A. A. (2011). *Acta Cryst.* D**67**, 355–367. 10.1107/S0907444911001314 PMC3069751 21460454

[bb12] Stein, N. (2008). *J. Appl. Cryst.* **41**, 641–643.

[bb13] St-Jean, M., Lafrance-Vanasse, J., Liotard, B. & Sygusch, J. (2005). *J. Biol. Chem.* **280**, 27262–27270. 10.1074/jbc.M502413200 15870069

[bb14] Studier, F. W. (2005). *Protein Expr. Purif.* **41**, 207–234. 10.1016/j.pep.2005.01.016 15915565

[bb2] Winn, M. D. *et al.* (2011). *Acta Cryst.* D**67**, 235–242.

